# Investigation of early-stage cardiovascular-kidney-metabolic syndrome on retinal structure and function evaluated by optical coherence tomography angiography

**DOI:** 10.3389/fmed.2025.1614321

**Published:** 2025-07-04

**Authors:** Yunyi Liang, Congbi Liang, Jianping Yang, Wanhua Li, Runyi Liang, Kun Li, Jingfeng Zhang, Peiqiong Lao, Junhua Zhu, Lin An, Zhaohao Liang, Chubin Ou

**Affiliations:** ^1^Health Management Center of The Sixth Affiliated Hospital, School of Medicine, South China University of Technology, Foshan, China; ^2^Department of Research and Development, Hangzhou Dianzi University, Hangzhou, China; ^3^Foshan Research Institute of the Hong Kong University of Science and Technology, Foshan, China; ^4^Department of Radiology, Guangdong Provincial People's Hospital (Guangdong Academy of Medical Sciences), Southern Medical University, Guangzhou, China

**Keywords:** cardiovascular-kidney-metabolic syndrome, optical coherence tomography angiography, biomarker, retinal thickness, vessel density (VD)

## Abstract

**Background:**

The retina serves as a non-invasive window to visualize systemic health, with optical coherence tomography angiography (OCTA) enabling simultaneous assessment of retinal structural thickness and vascular density. Suboptimal cardiovascular-kidney-metabolic (CKM) health status, defined by the early stage of CKM syndrome, may impact retinal neurovascular integrity, yet comprehensive OCTA-based evaluations remain limited and controversial. This study aimed to investigate retinal structural and vascular alterations in the early-stage CKM using OCTA and explore its potential as a biomarker tool.

**Methods:**

A cross-sectional study included 2,153 participants undergoing OCTA during health checks (2023–2024). Early-stage CKM (stages 1–2) is characterized by abnormal levels of glucose, lipid profile, uric acid, or blood pressure. Retinal thickness and vessel density in nine ETDRS grid regions were measured. Univariate and multivariate logistic regression analyses identified associations between early-stage CKM and OCTA parameters, adjusting for age and gender.

**Results:**

Early-stage CKM subjects (*n* = 1,843, 94.5%) exhibited significantly reduced retinal thickness and vessel density across multiple regions compared to healthy controls (all *p* < 0.05). Multivariate analysis revealed outer temporal thickness (OR = 0.986, *p* < 0.001), outer superficial vessel density (OR = 1.04, *p* = 0.020), and inner superior vessel density (OR = 0.962, *p* = 0.010) as independent predictors of early-stage CKM, alongside age and gender. Specific abnormalities, such as hypertension and diabetes, were associated with distinct regional decreases in thickness and density, suggesting a dose-dependent impact of glycemic dysregulation on retinal health.

**Conclusion:**

Early-stage CKM syndrome is associated with subtle retinal structural and vascular alterations detectable by OCTA, particularly within the macular layers. OCTA-derived biomarkers, including outer temporal thickness and macular vessel density, may serve as non-invasive tools for evaluating systemic health status, offering promise for the early detection of metabolic and cardiovascular risks.

## Background

The retina presents a distinct, non-invasive, and *in vivo* means of visualizing the vasculature and neural tissues of the human body. It functions as a window into overall health. The retinal nerve fibers are an extension of the axons in the central nervous system (CNS), and the retinal ganglion cells (RGCs) exhibit the typical characteristics of CNS neurons (1). The retinal blood vessels not only reflect the features and regulatory mechanisms of blood vessels throughout the body but also act as indicators of general wellbeing (2).

In the last few decades, research provided evidence of RGC degeneration and damage to the retinal nerve fiber layer (RNFL) in Alzheimer’s disease (AD), a neurodegenerative disorder (3). Additionally, pathological changes were found in the retinal vasculature in cases of stroke (cerebral hemorrhage and infarction) (4). Insights from numerous current studies have also confirmed the correlation between retinal images and the risks of dementia and stroke, which are related to neural and vascular abnormalities, respectively (5, 6). These studies have laid the groundwork for using retinal images to evaluate and predict systemic diseases.

Optical coherence tomography (OCT) offers cross-sectional details of the retina and choroid, while OCT angiography (OCTA) visualizes the vasculature and structure within specific single layers. These technologies can serve as biomarkers for neuronal pathways because of their detailed imaging of RGCs, the RNFL, the inner plexiform layer (IPL), and other neural layer structures (7). Meanwhile, CFP and OCTA can be used for studying vascular pathways (8). Various artificial intelligence methods have been developed to facilitate the use of retinal imaging in the diagnosis and quantitative analysis of both retinal disease (9–12) and systemic diseases (13–16).

OCTA studies investigating superficial vascular density in the eyes of patients with diabetes but no signs of diabetic retinopathy have yielded mixed results. While some studies observed decreased superficial vascular density in diabetic patients without retinopathy (17, 18), others observed no change in density (19, 20). In contrast, investigations into deep vascular density in the eyes of patients with diabetes without signs of diabetic retinopathy, compared to healthy controls, have yielded more consistent findings. Notably, in the majority of studies, a reduction in deep vascular density was reported in diabetic patients without retinopathy (21, 22).

Research efforts exploring the influence of systemic hypertension on OCTA parameters have also yielded somewhat inconsistent findings. Although the majority of studies indicate notable decreases in the superficial vascular density among hypertensive patients (23, 24), some did not observe a significant decrease (25).

Consistent findings from multiple studies indicate that obese adults, even those without diabetes or hypertension, exhibit decreased vascular density of the deep retinal layer (26). Wang et al. studied the association between retinal thickness and fundus blood flow density in chest pain patients with dyslipidemia. They found that the dyslipidemia group had significantly lower superficial vascular density in the inferior and temporal retinal regions than the control group, indicating a potential connection between dyslipidemia and retinal microvascular changes (27).

In this study, unlike previous studies, we focused on investigating differences in retinal structure and function on OCTA between healthy subjects and those with early-stage cardiovascular-kidney-metabolic syndrome. Cardiovascular-kidney-metabolic (CKM) health, first proposed by the American Heart Association, encompasses the clinical manifestations resulting from the pathophysiological interactions among metabolic risk factors, such as obesity and diabetes, chronic kidney disease, and the cardiovascular system (28). A CKM health framework has been proposed to facilitate early detection of suboptimal CKM health to enable timely intervention and slow disease progression (29). In the current study, we focused on early-stage CKM syndrome subjects. Specifically, early-stage CKM syndrome was defined by the presence of prediabetes, diabetes, or hypertension and any signs of abnormal levels of key metabolic and cardiovascular blood indicators, including glucose, triglyceride, total cholesterol, HDL, LDL, and uric acid. We aimed to identify whether OCTA imaging biomarkers can be used as an evaluation tool for predicting early CKM syndrome.

## Methods

### Data collection

**Figure 1:**
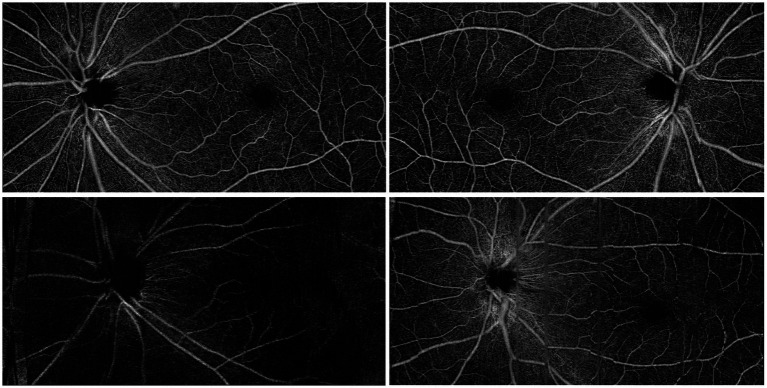
Top row: typical OCTA scan images. Bottom left: an excluded image due to low contrast; bottom right: an excluded image due to motion artifact.

A total of 2,153 participants receiving health check-ups with retinal fundus OCTA imaging between 2023 and 2024 in the Sixth Hospital of South China University of Technology were enrolled. Individuals with a known history of cardiovascular disease were excluded. Gender, age, lipid profile, blood pressure, glucose level, and uric acid were measured for all participants. Retinal imaging was performed using a Velite-C3000 SD-OCTA scanner, which features a 120 kHz scanning speed, OMAG algorithm, and eye-tracking technology to minimize motion artifacts during scanning. Three-dimensional (3D) scans covering a 12 mm x 6 mm area were obtained for both eyes, centered at the middle point between the macula and optic disc to ensure that both structures were captured. To ensure the quality of the study, we excluded images of low quality due to motion artifacts or low contrast. Two typical scan images are shown in Figure 1, along with examples of excluded images due to low contrast and motion artifacts.

We manually examined the retinal layer segmentation and corrected those obvious mistakes. The total thickness of the retina was then measured automatically by the vendor machine and reported according to the nine regions defined by the ETDRS grid, which are outer superior (SOM), inner superior (SIM), outer temporal (TOM), inner temporal (TIM), outer inferior (IOM), inner inferior (IIM), outer nasal (NOM), inner nasal (NIM), and center subfield (CSF). The vendor machine software features an automatic calculation of vessel density, which was based on a validated deep learning method described in a previous study (30). The area-averaged vessel density was also calculated according to the ETDRS zone.

### Grouping criteria

The normal range of blood biomarkers is defined as follows: LDL: <3.7 mmol/L; HDL: >1.0 mmol/L (male) or >1.2 mmol/L (female); triglycerides: <2.3 mmol/L; total cholesterol: <5.2 mmol/L; and blood uric acid: 0.21 ~ 0.42 mmol/L (male), or 0.15 ~ 0.36 mmol/L (female). Any value falling outside the normal range was classified as abnormal. For glucose levels, we further divided participants into diabetes and prediabetes groups. Prediabetes was defined in individuals without diabetes who meet any of the following criteria: fasting glucose level between 6.1–7 mmol/L, post-prandial glucose level between 7.8–11.1 mmol/L, or HbA1c > 5.7%. Hypertension was defined as systolic blood pressure > 140 mmHg, diastolic pressure >90 mmHg, or a prior diagnosis of hypertension. According to the definition provided by the American Heart Association (28), individuals with dyslipidemia, dysuricemia, diabetes, prediabetes, or hypertension are considered to have suboptimal CKM health (CKM syndrome stages 1–2).

### Statistical analysis

All variables were subjected to univariate comparisons between the low- and high-risk groups. For binary or categorical variables, either Fisher’s exact test or the chi-squared test was used. Continuous variables underwent normality assessment using the Shapiro–Wilk test, subsequent to which the Student’s *t*-test was applied for variables with normal distribution and the Mann–Whitney U-test for those in non-normal distribution. Only variables that were statistically significant and independent were included in the multivariate logistic regression. A multivariate logistic regression analysis was performed with a backward stepwise method after adjusting for confounding factors such as age and gender. Variable collinearity was assessed using Pearson’s correlation test. Multiple comparisons were corrected using the Bonferroni correction method. A *p*-value of less than 0.05 was deemed to indicate statistical significance. The aforementioned analyses were conducted utilizing SPSS software (IBM Corporation, United States).

## Results

### Demographic and baseline characteristics

Of the 2,153 subjects enrolled, 204 were excluded due to low image quality or missing data; as a result, a total of 1,949 participants were eventually included in the final analysis, with 1,843 (94.5%) classified as early-stage CKM syndrome based on abnormal blood biomarkers (glucose, lipid profile, uric acid, blood pressure, or diabetes status) and 106 (5.5%) as healthy controls. The mean age of the cohort was 59.2 ± 7.74 years, with women comprising 68.7%. The prevalence of abnormal biomarkers included total cholesterol (52.0%), triglycerides (41.1%), LDL (37.7%), hypertension (45.9%), diabetes (23.3%), HDL (23.1%), and uric acid (9.2%), as shown in Table 1.

**Table 1:** Baseline characteristics of the studied subjects.

Condition	Normal	Abnormal
Mean age	54.0 ± 9.72	59.5 ± 7.49
Female	45 (42.4%)	1,180 (64.0%)
Suboptimal health	106 (5.5%)	1843 (94.5%)
Abnormal LDL	1,214 (62.3%)	735 (37.7%)
Abnormal HDL	1,498 (76.9%)	451 (23.1%)
Abnormal triglyceride	1,147 (58.9%)	802 (41.1%)
Abnormal total cholesterol	935 (48.0%)	1,014 (52.0%)
Abnormal uric acid	1760 (90.8%)	180 (9.2%)
Diabetes	1,495 (76.7%)	454 (23.3%)
Hypertension	1,054 (54.1%)	895 (45.9%)

LDL, low density lipoprotein; HDL, high density lipoprotein.

### Univariate analysis: control vs. early-stage CKM groups

Early-stage CKM subjects exhibited significantly thinner retinal thickness in all nine ETDRS grid regions compared to healthy controls (all *p* < 0.05), with the most pronounced reductions in the temporal outer macula region (TOM, 269 ± 17.9 vs. 273 ± 15.3, *p* < 0.001) and superior inner macula (SIM, 317 ± 19.6 vs. 321 ± 15.6, p < 0.001), as shown in Table 2. Vascular density was also significantly lower in the early-stage CKM group across all regions, particularly within the superficial capillary plexus: superior outer macula (SOM, 27.0 ± 11.5 vs. 30.5 ± 12.2, *p* < 0.001) and nasal inner macula (NIM, 17.3 ± 12.9 vs. 20.8 ± 14.0, *p* < 0.001).

**Table 2:** Univariate analysis between the health and early stage CKM groups.

Variables	Health	Suboptimal CKM health	*p* value
	Mean ± SD (95% CI)	Mean ± SD (95% CI)	
Age	54.0 ± 9.71 (52.70–55.29)	59.9 ± 7.49 (59.31–59.80)	<0.001
Gender (female)	79.5%	68.2%	<0.001
Retinal thickness			
SOM	293 ± 15.1 (291.8–295.8)	290 ± 16.8 (289.8–290.9)	0.001
NOM	305 ± 14.9 (303.0–307.0)	299 ± 18.9 (299.1–300.4)	<0.001
IOM	280 ± 14.2 (278.5–282.3)	276 ± 15.7 (275.5–276.6)	<0.001
TOM	273 ± 15.3 (271.5–275.6)	269 ± 17.9 (268.8–269.9)	<0.001
SIM	321 ± 15.6 (319.8–323.9)	317 ± 19.6 (316.5–317.7)	<0.001
NIM	320 ± 18.0 (317.9–322.7)	315 ± 22.5 (314.9–316.3)	0.001
IIM	318 ± 15.0 (316.3–320.3)	313 ± 19.1 (313.0–314.3)	0.001
TIM	309 ± 16.7 (306.9–311.3)	304 ± 21.0 (304.1–305.4)	0.001
CSF	246 ± 18.6 (243.5–248.5)	243 ± 25.9 (242.4–244.1)	0.019
Vessel density			
SOM	30.5 ± 12.2 (28.88–32.14)	27.0 ± 11.5 (26.65–27.39)	<0.001
NOM	29.5 ± 14.1 (27.65–31.44)	26.0 ± 13.4 (25.64–26.50)	<0.001
IOM	28.9 ± 11.7 (27.35–30.46)	25.3 ± 11.4 (24.92–25.66)	<0.001
TOM	24.2 ± 12.3 (22.57–25.84)	21.5 ± 11.5 (21.13–21.87)	0.002
SIM	27.5 ± 13.8 (25.72–29.38)	23.2 ± 12.9 (22.83–23.66)	<0.001
NIM	20.8 ± 14.0 (19.01–22.74)	17.3 ± 12.9 (16.90–17.74)	<0.001
IIM	24.2 ± 13.5 (22.41–26.00)	20.4 ± 12.8 (19.99–20.82)	<0.001
TIM	19.3 ± 13.2 (18.16–21.69)	16.3 ± 11.9 (15.92–16.69)	<0.001
CSF	4.87 ± 7.21 (3.91–5.83)	3.28 ± 4.55 (3.13–3.43)	<0.001

SOM, outer superior; NOM, outer nasal; IOM, outer inferior; TOM, outer temporal; SIM, inner superior; NIM, inner nasal; IIM, inner inferior; TIM, inner temporal; CSF, central subfield.

### Multivariate logistic regression

After adjusting for confounding factors, thinner TOM thickness (OR = 0.986, 95% CI: 0.978–0.993, *p* < 0.001), lower SOM vascular density (OR = 1.04, 95% CI: 1.006–1.075, *p* = 0.020), lower SIM vascular density (OR = 0.962, 95% CI: 0.935–0.991, *p* = 0.010), older age (OR = 1.076, 95% CI: 1.058–1.095, *p* < 0.001), and female gender (OR = 0.506, 95% CI: 0.358–0.714, *p* < 0.001) were independently associated with suboptimal CKM health status, as shown in Table 3.

**Table 3:** Multivariate analysis between the health and early stage CKM groups.

Variables	Odds ratio (95% CI)	*p* value
TOM thickness	0.986 (0.978–0.993)	<0.001
SOM vessel density	1.04 (1.006–1.075)	0.020
SIM vessel density	0.962 (0.935–0.991)	0.010
Age	1.076 (1.058–1.095)	<0.001
Gender	0.506 (0.358–0.714)	<0.001

SOM, outer superior; TOM, outer temporal; SIM, inner superior.

### Subgroup analyses by abnormal biomarkers

We further performed a subgroup analysis based on different abnormal biomarkers or suboptimal CKM health status. The subgroup analysis involved separate two-group comparisons, where the total subjects were divided into healthy and abnormal groups according to specific blood biomarker levels.

For LDL and total cholesterol, no significant retinal thickness or vascular density differences were observed between normal and abnormal groups, while triglyceride abnormalities were associated with increased NIM and IIM vascular density (*p* < 0.05), and low HDL was associated with thinner SOM thickness (*p* = 0.025) and reduced vascular density in TOM, inferior inner macula (IIM), and TIM (*p* < 0.05), as shown in Tables 4–7.

**Table 4:** Univariate analysis between the normal and abnormal LDL groups.

Variables	Normal LDL (1214)	Abnormal LDL (753)	*p* value
	Mean±SD (95% CI)	Mean±SD (95% CI)	
Retinal thickness			
SOM	290 ± 17.3 (289.8–291.2)	290 ± 15.7 (289.8–291.4)	0.899
NOM	300 ± 19.4 (299.4–300.9)	299 ± 17.5 (298.9–300.7)	0.307
IOM	276 ± 16.1 (275.7–277.0)	276 ± 14.9 (275.4–277.0)	0.624
TOM	270 ± 18.3 (263.3–270.7)	268 ± 17.0 (268.1–269.8)	0.175
SIM	317 ± 19.9 (316.5–318.0)	317 ± 18.6 (316.6–318.5)	0.638
NIM	315 ± 22.8 (314.7–316.5)	316 ± 21.4 (315.2–317.4)	0.502
IIM	313 ± 18.9 (313.2–314.7)	313 ± 18.9 (313.9–314.8)	0.777
TIM	305 ± 21.2 (304.2–305.8)	304 ± 20.0 (303.9–306.0)	0.512
CSF	243 ± 25.9 (242.5–244.6)	243 ± 24.9 (241.9–244.5)	0.774
Vessel density			
SOM	27.1 ± 11.7 (26.67–27.60)	27.3 ± 11.5 (26.76–27.93)	0.640
NOM	26.3 ± 13.6 (25.83–26.91)	26.0 ± 13.2 (25.41–26.77)	0.503
IOM	25.5 ± 11.5 (25.05–25.96)	25.4 ± 11.3 (24.88–26.05)	0.878
TOM	21.7 ± 11.6 (21.29–22.22)	21.4 ± 11.4 (20.89–22.07)	0.497
SIM	23.4 ± 13.0 (22.94–23.97)	23.5 ± 12.9 (22.86–24.19)	0.922
NIM	17.5 ± 13.0 (17.05–18.08)	17.4 ± 13.0 (16.78–18.12)	0.833
IIM	20.7 ± 12.9 (20.19–21.21)	20.4 ± 12.7 (19.82–21.12)	0.630
TIM	16.5 ± 12.1 (16.09–17.05)	16.4 ± 11.8 (15.79–17.01)	0.925
CSF	3.42 ± 4.95 (3.23–3.62)	3.28 ± 4.39 (3.05–3.50)	0.811

SOM, outer superior; NOM, outer nasal; IOM, outer inferior; TOM, outer temporal; SIM, inner superior; NIM, inner nasal; IIM, inner inferior; TIM, inner temporal; CSF, central subfield.

**Table 5:** Univariate analysis between the normal and abnormal HDL groups.

Variables	Normal HDL (1498)	Abnormal HDL (451)	*p* value
	Mean±SD (95% CI)	Mean±SD (95% CI)	
Retinal thickness			
SOM	290 ± 16.8 (290.3–291.5)	289 ± 16.3 (288.4–290.5)	0.025
NOM	300 ± 19.0 (299.5–300.9)	299 ± 17.8 (298.4–300.7)	0.484
IOM	276 ± 15.7 (275.9–277.1)	275 ± 15.5 (274.7–276.7)	0.156
TOM	269 ± 17.4 (269.0–270.3)	269 ± 19.2 (268.3–270.9)	0.917
SIM	317 ± 19.8 (316.9–318.3)	316 ± 18.2 (315.3–317.7)	0.073
NIM	316 ± 22.6 (315.4–317.0)	314 ± 21.3 (313.2–315.9)	0.082
IIM	314 ± 19.5 (313.3–314.7)	313 ± 16.7 (312.3–314.5)	0.386
TIM	305 ± 21.1 (304.4–305.9)	304 ± 19.5 (303.0–305.5)	0.250
CSF	243 ± 25.9 (242.7–244.5)	242 ± 24.1 (241.1–244.3)	0.432
Vessel density			
SOM	27.4 ± 11.6 (27.04–27.87)	26.4 ± 11.6 (25.66–27.18)	0.023
NOM	26.4 ± 13.3 (25.96–26.92)	25.6 ± 13.7 (24.77–26.57)	0.115
IOM	25.6 ± 11.4 (25.25–26.07)	24.9 ± 11.5 (24.17–25.67)	0.074
TOM	21.9 ± 11.6 (21.54–22.37)	20.6 ± 11.2 (19.92–21.39)	0.004
SIM	23.6 ± 12.9 (23.19–24.12)	22.9 ± 13.0 (22.06–23.76)	0.124
NIM	17.6 ± 13.0 (17.20–18.13)	17.0 ± 13.2 (16.17–17.89)	0.093
IIM	20.8 ± 12.8 (20.39–21.30)	19.8 ± 12.8 (19.01–20.69)	0.028
TIM	16.7 ± 12.1 (16.32–17.18)	15.6 ± 11.8 (14.92–16.46)	0.020
CSF	3.39 ± 4.71 (3.22–3.56)	3.29 ± 4.86 (2.97–3.60)	0.052

SOM, outer superior; NOM, outer nasal; IOM, outer inferior; TOM, outer temporal; SIM, inner superior; NIM, inner nasal; IIM, inner inferior; TIM, inner temporal; CSF, central subfield.

**Table 6:** Univariate analysis between the normal and abnormal triglyceride groups.

Variables	Normal triglyceride (1147)	Abnormal triglyceride (802)	*p* value
	Mean±SD (95% CI)	Mean±SD (95% CI)	
Retinal thickness			
SOM	291 ± 17.0 (290.0–291.4)	290 ± 16.3 (289.5–291.1)	0.747
NOM	300 ± 19.2 (299.4–301.0)	300 ± 18.0 (298.9–300.7)	0.784
IOM	276 ± 15.7 (275.8–277.1)	276 ± 15.5 (275.3–276.8)	0.808
TOM	270 ± 16.9 (269.3–270.6)	269 ± 19.1 (268.2–270.1)	0.655
SIM	317 ± 20.7 (316.4–318.1)	317 ± 17.4 (316.7–318.4)	0.056
NIM	315 ± 23.3 (315.5–316.4)	316 ± 20.2 (315.4–317.4)	0.022
IIM	313 ± 20.0 (312.8–314.4)	314 ± 17.2 (313.4–315.1)	0.025
TIM	305 ± 20.8 (304.2–305.9)	304 ± 20.7 (303.8–305.9)	0.155
CSF	243 ± 28.2 (242.4–244.8)	243 ± 21.1 (242.1–244.2)	0.483
Vessel density			
SOM	26.9 ± 11.6 (26.47–27.42)	27.6 ± 11.6 (27.03–28.17)	0.067
NOM	25.9 ± 13.3 (25.37–26.47)	26.7 ± 13.5 (26.09–27.42)	0.067
IOM	25.2 ± 11.4 (24.76–25.70)	25.8 ± 11.5 (25.29–26.42)	0.082
TOM	21.5 ± 11.6 (21.06–22.01)	21.8 ± 11.5 (21.26–22.38)	0.451
SIM	23.1 ± 12.9 (22.65–23.71)	23.9 ± 13.0 (23.28–24.55)	0.079
NIM	17.0 ± 12.8 (16.53–17.58)	18.2 ± 13.3 (17.53–18.84)	0.015
IIM	20.2 ± 12.7 (19.68–20.72)	21.2 ± 12.9 (20.58–21.84)	0.015
TIM	16.3 ± 12.1 (15.82–16.80)	16.8 ± 12.0 (16.20–17.37)	0.123
CSF	3.28 ± 4.88 (3.09–3.48)	3.49 ± 4.56 (3.27–3.71)	0.012

SOM, outer superior; NOM, outer nasal; IOM, outer inferior; TOM, outer temporal; SIM, inner superior; NIM, inner nasal; IIM, inner inferior; TIM, inner temporal; CSF, central subfield.

**Table 7:** Univariate analysis between the normal and abnormal cholesterol groups.

Variables	Normal total cholesterol (935)	Abnormal total cholesterol (1014)	*p* value
	Mean±SD (95% CI)	Mean±SD (95% CI)	
Retinal thickness			
SOM	290 ± 17.5 (288.2–290.8)	291 ± 15.9 (290.4–298.8)	0.207
NOM	300 ± 19.3 (299.2–300.8)	300 ± 18.1 (299.5–300.6)	0.395
IOM	276 ± 15.7 (275.5–276.9)	276 ± 15.6 (275.7–277.1)	0.851
TOM	269 ± 17.7 (269.0–270.6)	269 ± 17.9 (268.7–270.2)	0.726
SIM	317 ± 19.9 (316.0–317.8)	317 ± 19.0 (316.9–318.6)	0.362
NIM	315 ± 23.5 (313.9–316.0)	317 ± 21.1 (315.7–317.6)	0.126
IIM	313 ± 18.8 (312.9–314.6)	314 ± 19.0 (313.2–314.9)	0.901
TIM	304 ± 21.3 (303.8–305.7)	305 ± 20.3 (304.3–306.1)	0.978
CSF	243 ± 25.4 (242.1–244.4)	243 ± 25.6 (242.5–244.7)	0.626
Vessel density			
SOM	27.0 ± 11.9 (26.47–27.54)	27.4 ± 11.3 (26.91–27.91)	0.268
NOM	26.1 ± 13.7 (25.55–26.79)	26.3 ± 13.1 (25.78–26.93)	0.620
IOM	25.3 ± 11.6 (24.77–25.82)	25.6 ± 11.3 (25.18–26.16)	0.284
TOM	21.6 ± 11.7 (21.11–22.16)	21.6 ± 11.4 (21.17–22.17)	0.749
SIM	23.4 ± 13.1 (22.84–24.02)	23.5 ± 12.8 (22.97–24.09)	0.707
NIM	17.4 ± 13.1 (16.81–18.00)	17.6 ± 12.9 (17.06–18.19)	0.414
IIM	20.6 ± 13.0 (20.01–21.18)	20.6 ± 12.7 (20.08–21.19)	0.745
TIM	16.5 ± 12.3 (16.03–17.14)	16.4 ± 11.8 (15.92–16.95)	0.884
CSF	3.50 ± 5.19 (3.27–3.73)	3.24 ± 4.29 (3.06–3.43)	0.846

SOM, outer superior; NOM, outer nasal; IOM, outer inferior; TOM, outer temporal; SIM, inner superior; NIM, inner nasal; IIM, inner inferior; TIM, inner temporal; CSF, central subfield.

Elevated uric acid was correlated with thinner superior outer macula (SOM) and inferior outer macula (IOM) thickness (*p* < 0.05) and higher central subfield (CSF) vascular density (*p* = 0.032), a finding inconsistent with other biomarkers, as shown in Table 8.

**Table 8:** Univariate analysis between the normal and abnormal uric acid groups.

Variables	Normal uric acid (1760)	Abnormal uric acid (180)	*p* value
	Mean±SD (95% CI)	Mean±SD (95% CI)	
Retinal thickness			
SOM	290 ± 16.4 (290.2–291.3)	288 ± 19.1 (286.5–290.4)	0.034
NOM	300 ± 18.7 (299.5–300.7)	299 ± 18.7 (297.3–301.1)	0.414
IOM	276 ± 15.5 (275.9–276.9)	274 ± 17.1 (273.2–276.7)	0.031
TOM	269 ± 17.4 (269.1–270.2)	269 ± 21.1 (266.9–271.3)	0.488
SIM	317 ± 19.2 (316.9–318.2)	316 ± 21.0 (313.7–318.0)	0.753
NIM	316 ± 21.8 (315.2–316.6)	315 ± 26.9 (312.3–317.8)	0.620
IIM	314 ± 18.8 (313.3–314.5)	313 ± 20.5 (311.4–315.6)	0.623
TIM	305 ± 20.6 (304.4–305.7)	304 ± 22.0 (302.2–306.7)	0.434
CSF	243 ± 25.7 (242.5–244.2)	243 ± 24.0 (241.2–246.2)	0.174
Vessel density			
SOM	27.1 ± 11.6 (26.81–27.58)	27.4 ± 11.9 (26.18–28.62)	0.644
NOM	26.2 ± 13.4 (25.80–26.68)	26.4 ± 13.8 (25.05–27.88)	0.756
IOM	25.4 ± 11.4 (25.08–25.83)	25.8 ± 12.1 (24.57–27.05)	0.573
TOM	21.5 ± 11.5 (21.20–21.96)	22.3 ± 11.9 (21.11–23.56)	0.239
SIM	23.4 ± 12.9 (22.96–23.81)	24.4 ± 13.3 (23.04–25.76)	0.137
NIM	17.4 ± 13.0 (17.00–17.86)	18.4 ± 13.6 (17.01–19.81)	0.281
IIM	20.5 ± 12.7 (20.10–20.94)	21.5 ± 13.3 (20.14–22.88)	0.209
TIM	16.4 ± 12.0 (16.02–16.81)	17.3 ± 12.5 (16.09–18.65)	0.211
CSF	3.32 ± 4.72 (3.16–3.47)	3.85 ± 5.03 (3.34–4.37)	0.032

SOM, outer superior; NOM, outer nasal; IOM, outer inferior; TOM, outer temporal; SIM, inner superior; NIM, inner nasal; IIM, inner inferior; TIM, inner temporal; CSF, central subfield.

Table 9 reveals that hypertensive subjects showed reduced retinal thickness in all regions (*p* < 0.05) and lower vascular density in SOM, the nasal outer macula (NOM), and the temporal inner macula (TIM) (*p* < 0.05).

**Table 9:** Univariate analysis between the normal and hypertension groups.

Variables	Normal (1054)	Hypertension (895)	*p* value
	Mean±SD (95% CI)	Mean±SD (95% CI)	
Retinal thickness			
SOM	292 ± 17.0 (291.5–292.9)	288 ± 16.1 (287.8–289.4)	<0.001
NOM	302 ± 16.8 (301.3–302.8)	297 ± 20.5 (296.7–298.6)	<0.001
IOM	277 ± 15.4 (276.7–278.0)	275 ± 15.9 (274.4–275.8)	<0.001
TOM	270 ± 17.7 (269.6–271.1)	268 ± 17.9 (267.9–269.6)	0.005
SIM	318 ± 20.3 (317.8–319.6)	315 ± 18.2 (314.9–316.6)	<0.001
NIM	316 ± 22.5 (315.9–317.8)	314 ± 22.1 (313.6–315.7)	0.001
IIM	314 ± 19.1 (313.9–315.5)	313 ± 18.7 (312.1–313.8)	<0.001
TIM	306 ± 20.4 (305.0–306.7)	304 ± 21.2 (303.0–304.9)	0.009
CSF	244 ± 26.5 (242.9–245.1)	242 ± 24.3 (241.6–243.9)	0.091
Vessel density			
SOM	27.8 ± 11.8 (27.35–28.35)	26.4 ± 11.4 (25.92–26.98)	<0.001
NOM	26.7 ± 13.6 (26.12–27.27)	25.7 ± 13.2 (25.13–26.36)	0.038
IOM	25.8 ± 11.6 (25.36–26.35)	25.0 ± 11.3 (24.52–25.58)	0.042
TOM	22.0 ± 11.7 (21.51–22.50)	21.2 ± 11.4 (20.70–21.77)	0.056
SIM	24.1 ± 13.1 (23.58–24.69)	22.0 ± 12.8 (22.11–23.30)	0.001
NIM	17.9 ± 13.1 (17.34–18.46)	17.0 ± 12.9 (16.46–17.66)	0.054
IIM	20.9 ± 12.9 (20.41–21.51)	20.2 ± 12.6 (19.62–20.80)	0.099
TIM	16.9 ± 12.2 (16.42–17.46)	15.9 ± 11.8 (15.44–16.54)	0.017
CSF	3.51 ± 4.86 (3.30–3.72)	3.25 ± 4.61 (2.99–3.41)	0.012

SOM, outer superior; NOM, outer nasal; IOM, outer inferior; TOM, outer temporal; SIM, inner superior; NIM, inner nasal; IIM, inner inferior; TIM, inner temporal; CSF, central subfield.

As for prediabetes and diabetes, both groups exhibited progressive decreases in retinal thickness and vascular density compared with the normal group, with the most severe reductions in diabetes (e.g., SOM vascular density: 24.8 ± 11.0 in diabetes vs. 28.8 ± 11.6 in normal, *p* < 0.001), as shown in Table 10.

**Table 10:** Univariate analysis between the normal and diabetes groups.

Variables	Normal glucose (575)	Prediabetes (920)	Diabetes (454)	*p* value
	Mean ± SD (95% CI)	Mean ± SD (95% CI)	Mean ± SD (95% CI)	
Retinal thickness				
SOM	291 ± 16.4 (290.7–292.7)	290 ± 15.8 (289.5–291.0)	289 ± 18.8 (288.5–290.9)	0.002
NOM	301 ± 17.6 (300.4–302.4)	299 ± 18.6 (298.6–300.3)	299 ± 20.3 (298.2–300.8)	0.009
IOM	277 ± 14.7 (276.4–278.1)	275 ± 15.4 (275.2–276.7)	275 ± 17.3 (274.8–277.0)	0.004
TOM	270 ± 15.6 (269.2–271.1)	269 ± 17.3 (268.8–270.3)	269 ± 21.3 (267.7–270.4)	0.077
SIM	319 ± 18.3 (318.1–320.3)	317 ± 19.3 (316.2–318.0)	315 ± 20.7 (314.3–317.0)	<0.001
NIM	317 ± 20.3 (316.7–319.1)	315 ± 22.3 (314.3–316.4)	314 ± 24.5 (312.7–315.9)	<0.001
IIM	315 ± 17.5 (314.3–316.4)	313 ± 19.2 (312.8–314.5)	312 ± 20.1 (311.2–313.9)	<0.001
TIM	306 ± 18.9 (305.4–307.6)	304 ± 21.6 (303.4–305.4)	304 ± 21.1 (302.8–305.6)	<0.001
CSF	244 ± 23.5 (242.9–245.6)	243 ± 25.2 (242.3–244.6)	242 ± 28.4 (240.5–244.2)	0.015
Vessel density				
SOM	28.8 ± 11.6 (28.16–29.51)	27.4 ± 11.6 (26.87–27.93)	24.8 ± 11.0 (24.10–25.53)	<0.001
NOM	27.8 ± 13.7 (27.08–28.67)	26.3 ± 13.3 (25.75–26.96)	24.0 ± 13.1 (23.22–24.92)	<0.001
IOM	26.9 ± 11.6 (26.24–27.59)	25.5 ± 11.5 (25.00–26.04)	23.6 ± 11.1 (22.92–24.36)	<0.001
TOM	23.0 ± 11.7 (22.38–23.74)	21.6 ± 11.6 (21.12–22.18)	19.8 ± 11.0 (19.18–20.60)	<0.001
SIM	25.2 ± 13.3 (24.44–25.98)	23.5 ± 12.9 (22.93–24.11)	21.2 ± 12.3 (20.44–22.04)	<0.001
NIM	18.9 ± 13.6 (18.15–19.73)	17.5 ± 13.0 (16.97–18.16)	15.6 ± 12.1 (1487–16.45)	<0.001
IIM	21.9 ± 13.2 (21.19–22.72)	20.7 ± 12.7 (20.12–21.28)	18.7 ± 12.3 (17.98–19.57)	<0.001
TIM	18.0 ± 12.5 (17.28–18.74)	16.5 ± 12.0 (15.97–17.07)	14.6 ± 11.1 (13.88–15.32)	<0.001
CSF	3.87 ± 5.34 (3.56–4.18)	3.31 ± 4.69 (3.10–3.53)	2.85 ± 3.37 (2.60–3.11)	<0.001

SOM, outer superior; NOM, outer nasal; IOM, outer inferior; TOM, outer temporal; SIM, inner superior; NIM, inner nasal; IIM, inner inferior; TIM, inner temporal; CSF, central subfield.

## Discussion

This study demonstrates that early-stage CKM syndrome is associated with widespread retinal structural and vascular changes. To our knowledge, this is the first study that examines the relationship between CKM syndrome and retinal structure and function. The most consistent findings were reduced retinal thickness and vascular density across multiple macular regions, particularly in the superior and temporal regions. These findings align with prior research linking systemic health to retinal microvascular dysfunction (1, 2). Given that aging is associated with vessel density reduction (31) and thickness reduction (32) and that men tend to exhibit lower vessel density (33). We further performed a multivariate analysis to adjust for confounding factors such as age and gender. The multivariate analysis highlighted the outer temporal region thickness and the outer and inner superior vascular density as potential independent biomarkers, even after adjusting for age and gender, suggesting that OCTA can detect early retinal changes even in asymptomatic individuals.

Research on superficial vascular density in diabetes patients without signs of diabetic retinopathy has produced mixed results, with some studies reporting decreased vascular density (9, 34), while others found no significant changes (10, 35). Studies on deep vascular density showed more consistent results of decreased vascular density. In our study, we observed significantly reduced retinal thickness and vessel density across almost all ETDRS regions. Furthermore, we observed a gradient change from normal to prediabetes to diabetes, highlighting the dose-dependent impact of glycemic dysregulation on retinal structure and function.

Research on the effect of systemic hypertension on OCTA parameters has led to somewhat conflicting results. Although the majority of studies have reported a significant reduction in hypertensive patients (23, 24) in both superficial and deep vascular density, some reported non-significant change (25). In our study, we observed a similar trend that almost all ETDRS regions showed a significant reduction in thickness and vascular density.

Retinal structure and function changes vary according to the lipid profile. Low HDL, a marker of impaired lipid metabolism, was associated with focal retinal thinning (outer superior) and vascular loss (outer superior, outer temporal, inner temporal, and inner inferior), potentially reflecting endothelial injury caused by dyslipidemia, which is consistent with Wang’s study (27), who also reported significantly reduced superficial vascular density in the inferior and temporal regions of patients in the dyslipidemia group. In our study, we further categorized dyslipidemia into four subgroups and found that abnormal HDL and triglyceride groups showed more significant changes in thickness and vessel density, while abnormal LDL and total cholesterol groups showed no significant change. Further studies are needed to elucidate the different effects of lipid substances on retinal function.

Yang et al. found that high uric acid level was associated with lower vessel density in men (36). However, in our study, we observed somewhat contradictory results, with vessel density showing an increasing trend in the dysuricemia group, although most quadrants were not statistically significant. This unexpected increase in vascular density with dysuricemia may indicate compensatory vasodilation, though further research is needed to clarify this relationship.

In our study, after adjusting for age and gender, the only significant changes in retinal thickness and vascular density were observed in the temporal and superior regions. A study by Lin et al. revealed that the temporal and superior regions are more vulnerable in patients with type 2 diabetes (37). Similar findings were also reported in the literature for the severity of diabetic retinopathy, where total thickness in the temporal and superior regions showed the most prominent change related to DR severity (38). A histologic study conducted by Kern et al., in an animal model of diabetes mellitus (DM), found that vascular disorders were more common in the superior temporal retina compared to the inferior nasal retina (39). One possible explanation for this phenomenon is that the temporal side of the retina usually has the lowest vessel density among the four quadrants because it lies furthest away from the optic disc region and thus is more susceptible to ischemia or hypoxia.

Retinal alterations in early-stage CKM likely result from systemic processes such as inflammation, oxidative stress, and endothelial dysfunction, which affect both neural and vascular tissues. The retina, as an extension of the central nervous system, is highly susceptible to metabolic stress, making OCTA’s ability to visualize both structural (thickness) and functional (vascular density) changes critical for early detection of systemic health decline. Therefore, OCTA-derived biomarkers may serve as non-invasive tools to assess suboptimal CKM health, particularly in populations at risk for diabetes or hypertension. The identification of retinal regions, such as outer nasal and outer superior, as key sites of change could guide targeted screening strategies. However, longitudinal studies are needed to validate these markers for predicting future cardiovascular or metabolic events.

### Limitations

This cross-sectional study cannot establish causality, and the single-center sample may limit generalizability. Additionally, the definition of early-stage CKM included multiple biomarkers, making it difficult to isolate individual effects. Only the total retina thickness and full retinal plexus vessel density were analyzed in the current study, while layer-specific thickness and plexus-specific vessel density were not analyzed. Future research should use longitudinal designs and mechanistic analyses to dissect the interplay between specific systemic abnormalities and retinal pathology.

## Conclusion

Early-stage CKM syndrome is associated with detectable retinal structural and vascular changes via OCTA, particularly in the macular region. These findings support OCTA as a promising tool for evaluating systemic health status, with potential for early intervention in the early stages of metabolic and cardiovascular disease.

## Data Availability

The original contributions presented in the study are included in the article/supplementary material, further inquiries can be directed to the corresponding authors.
